# Characterization of Multilayer Structure-Graded Dental Zirconias

**DOI:** 10.3390/bioengineering13040462

**Published:** 2026-04-14

**Authors:** Ragai-Edward Matta, Renan Belli, Katrin Hurle, Arulraj Sangarapillai, Oleksandr Sednyev, Manfred Wichmann, Lara Berger

**Affiliations:** 1 Department of Prosthodontics, University Hospital Erlangen, Glückstrasse 11, 91054 Erlangen, Germanyoleksandr.sednyev@uk-erlangen.de (O.S.); claudia.ehrhardt@uk-erlangen.de (M.W.); lara.berger@uk-erlangen.de (L.B.); 2 Research Laboratory for Dental Biomaterials, Department for Operative Dentistry and Periodontology, University Hospital Erlangen, Glückstrasse 11, 91054 Erlangen, Germany; renan.belli@fau.de; 3 GeoZentrum Nordbayern, Mineralogy, Friedrich-Alexander-Universität Erlangen-Nürnberg (FAU), Schlossgarten 5a, 91054 Erlangen, Germany; katrin.hurle@fau.de

**Keywords:** functionally graded biomaterials, multilayer zirconia, yttria-stabilized zirconia, Rietveld refinement, flexural strength, fracture toughness, phase transformation

## Abstract

Multilayer zirconias have recently been introduced as dental biomaterials to combine improved translucency with sufficient mechanical reliability by implementing yttria-driven gradients in phase composition. Such materials can be considered functionally graded ceramics, where local phase stabilization influences strength and crack resistance. However, manufacturer-specific gradient profiles and their structure–property relationships remain insufficiently characterized. This study investigated two commercially available multilayer zirconias with distinct gradient concepts: IPS e.max^®^ ZirCAD Prime (continuous gradient) and KATANA™ Zirconia YML (stepwise gradient). Ten equidistant sections along the blank height were analyzed using quantitative X-ray diffraction and Rietveld refinement to quantify zirconia phase fractions and estimate local Y_2_O_3_ content. Mechanical behavior was evaluated by biaxial flexural strength testing (ball-on-three-balls method) and fracture toughness testing using the chevron-notched beam technique. Both materials exhibited pronounced yttria- and phase-dependent gradients consistent with their reported layer designs. Regions with increased yttria content showed higher t″ fractions and reduced fracture toughness and strength, whereas deeper regions displayed increased mechanical performance associated with higher fractions of transformable tetragonal phase. These findings emphasize that multilayer zirconias exhibit spatially dependent mechanical properties, which should be considered in biomaterial selection and restoration design, particularly when balancing aesthetic demands and fracture resistance.

## 1. Introduction

Recent advancements in dental zirconia have introduced multilayer, structure-graded materials. This first step toward reproducing properties ubiquitous to natural materials—in this case structure gradient—integrates polycrystalline zirconia grains with different crystalline phases ordered in a unidirectional fractional transition within a single monolithic machinable blank [1]. To achieve this, manufacturers needed not to abandon the hitherto processing route of uniaxial (and subsequent cold-isostatic) powder pressing [2], but adapt the stage of powder pouring to incorporate spray-dried zirconia feedstocks containing primary particles stabilized with different Y_2_O_3_ amounts (usually between 3 and 6 mol%). Intervaled pouring leads to stepwise layered structures with defined transitions [3], whereas continuous mixing of different feedstocks during pouring achieves a continuous gradient along the pressing direction. The stepwise and gradient profiles and their phase ranges thus depend respectively on the pouring parameters and powders used, so that the resulting structure along the height of a pressed blank is process- and product-specific. Information about the pouring processes used by different manufacturers constitutes here the critical safeguarded industrial secret and remains generally inaccessible.

The structure profile is of utmost importance because it will determine both optical and mechanical properties simultaneously. Because these properties are mutually exclusive in dental zirconias [4], the actual profile consists of a double gradient in opposing directions. Powders containing higher Y_2_O_3_ concentration will tend to stabilize more of the higher-symmetry phases (t″, low tetragonality, near-cubic) [5], which are less birefringent, and induce a coarser grain size distribution with less surface refractive grain boundaries [6]. Both features increase light transmittance through the material. Mechanical toughening, on the other hand, increases inversely, namely with the amount of the less symmetrical tetragonal phase (t, high tetragonality), which is the metastable phase transformable to the monoclinic *m*-phase upon crack growth [7,8].

Recent studies have confirmed that multilayer zirconia systems exhibit pronounced layer-dependent differences in microstructure, yttria content and phase composition, which strongly influence both optical and mechanical properties of dental restorations. In particular, gradient zirconia systems have been developed to integrate high translucency with sufficient mechanical strength within a single restoration. Investigations by Gomes et al. [9] demonstrated that variations in yttria content across multilayer zirconia significantly influence both translucency and flexural strength. Similarly, Maharishi et al. [10] reported that strength-graded zirconia systems are designed to combine highly translucent layers with mechanically stronger regions, enabling improved functional and aesthetic performance in monolithic restorations. Furthermore, Cho et al. [11] showed that microstructural characteristics and optical behavior in multilayer zirconia are closely linked to yttria concentration and grain size distribution. In addition to compositional effects, processing parameters may further influence the resulting material properties. For example, Alatrash and Nalbant [12] demonstrated that sintering conditions significantly affect the microstructure and mechanical performance of multilayer zirconia ceramics. Likewise, Ham et al. [13] reported that different multilayer zirconia systems exhibit distinct variations in flexural strength and surface characteristics depending on the composition and gradient design. The phase composition of zirconia ceramics is commonly characterized using X-ray diffraction (XRD), which allows for the identification of crystalline phases and the quantification of phase fractions in zirconia-based materials, as demonstrated in recent studies employing XRD for the structural characterization of biomedical ceramics [14]. The aim of this study is the gradient characterizations of two commercially available “multilayered” zirconia blanks (one continuous gradient and one stepwise gradient) in regard to their phase structure, Y_2_O_3_ content and the mechanical behavior by means of strength and fracture toughness testing.

## 2. Materials and Methods

### 2.1. Materials

Two commercial materials were selected for this study due to their different gradient profiles as disclosed by the manufacturers. IPS e.max^®^ ZirCAD Prime (Ivoclar, Schaan, Lichtenstein) is alleged by the manufacturer to have an “incisal zone” of ~20% of the blank’s height, with a continuous “transition zone” of ~25% of the blank’s height merging into a “dentin zone” that composes the rest (~55%) of the blank. According to the manufacturer, the incisal zone’s main phase content is 5 mol% Y_2_O_3_-stabilized zirconia, and the dentin zone’s composition is based on 3 mol% Y_2_O_3_-stabilized zirconia. KATANA^TM^ Zirconia YML (Kuraray Noritake, Tokyo, Japan), on the other hand, exhibits a stepwise gradient profile, in which the “enamel” layer fills ~35% of the height, followed by two intermediate layers, “body 1” and “body 2”, of 15% relative thickness each, that merge in the “body 3” layer of 35% height. Although the manufacturer does not specify the composition of each layer, it states that the “enamel” layer reaches a flexural strength of 750 MPa, increasing to 1000 MPa within the body 1 and body 2 intermediate layers and reaching up to 1100 MPa in the body 3 layer.

### 2.2. Quantitative X-Ray Diffraction (QXRD) Analysis

The slices were characterized by X-ray diffraction (XRD) at a D8 diffractometer (Bruker AXS, Karlsruhe, Germany) equipped with a 9-fold sample changer. The following parameters were applied: range, 25–90° 2θ; step size, 0.011° 2θ; integration time, 1 s; radiation, copper K_α_; generator settings: 40 kV, 40 mA; divergence slit, 0.3°; detector: LynxEye. The slices were measured in their bulk form and fixed into special sample holders using plasticine. Each slice was analyzed twice.

Quantitative evaluation of the diffraction patterns was performed by Rietveld refinement with software TOPAS V5 (Bruker AXS, Karlsruhe, Germany). The structure models for the refinement of the crystalline ZrO_2_ phases were obtained from the ICSD database (Table 1). The refinement approach established for different non-graded ZrO_2_(Y) materials was applied [15]: The structure for tetragonal ZrO_2_(Y) was inserted twice and refined to different lattice parameters. It was further tested whether the additional insertion of a cubic structure would result in improvement of the fit. A Chebychev polynomial of the 5th order was used for the background contribution.

As in [15], the Y_2_O_3_ content of the two tetragonal ZrO_2_(Y) phases was estimated from the lattice parameters obtained by Rietveld refinement using the equation from Krogstad et al. [17] (Equation (1)).

(1) $${YO}_{1.5}[mol\%]\;=\;\frac{1.02311-\frac{c}{a_{t}}}{0.001498}$$

The ratio of the lattice parameters $\frac{c}{a_{t}}$ is the tetragonality, with $a_{t}\;=\;\sqrt{2}a$. The Y_2_O_3_ content [mol%] is then obtained by Equation (2) [18].

(2) $$Y_{2}O_{3}[mol\%]\;=\;\frac{{YO}_{1.5}[mol\%]/100}{2-Y_{1.5}[mol\%]/100}\cdot 100$$

The bulk Y_2_O_3_ content of each slice analyzed was then estimated from the weight fractions and Y_2_O_3_ contents of both t and t″.

### 2.3. Biaxial Flexural Strength Testing

**Figure 1:**
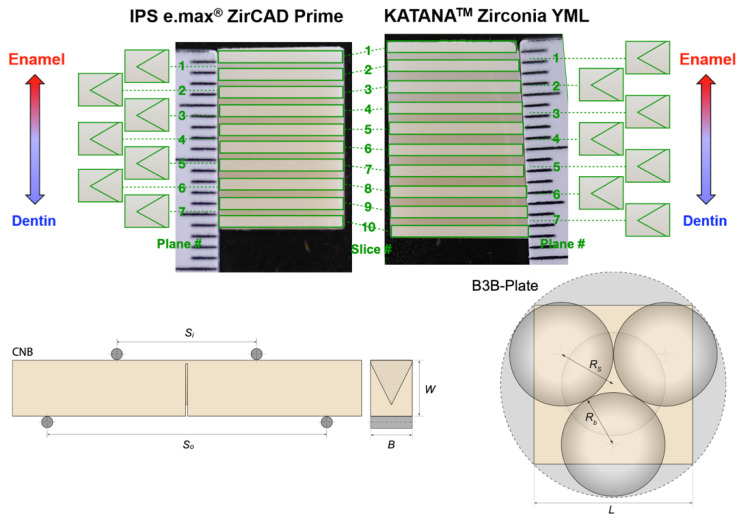
Scheme showing the cross-section of the blanks after sintering (scale in millimeters). Left-hand side: IPS e.max^®^ ZirCAD Prime. Right-hand side: KATANA^TM^ Zirconia YML. In green contours are the overlayered slices from where the biaxial strength plates were cut from (slices #1 to #10) and the CNB toughness specimen cross-sections (planes #1 to #7). Below are the configurations of the mechanical tests (CNB for fracture toughness) and (B3B-plate) for biaxial strength tests.

Along the height of the blanks of the two materials, oversized plates of 12 mm × 12 mm in sides and 1 mm in thickness (plus 20% linear shrinkage) were cut with a low-speed saw to obtain equidistant slices in 10 different heights, according to the scheme shown in Figure 1. Note that the height of the blanks is different for the two materials, so that the spacing for KATANATM Zirconia YML was slightly wider. Sintering was performed according to the manufacturer’s instructions. In total, 222 specimens were obtained, 22 per plane for each material.

The biaxial strength of each individual specimen obtained for each level (slices #1 to #10) was measured using the ball-on-three-balls (B3B) configuration using the Leoben jig [19] and stress solutions. The stress at fracture (σ_f_,_B3B_) is calculated at the tensile side of the specimen from the maximum force F_max_ [19]:

(3) $$\sigma_{f,B3B}\;=\;\delta \frac{F_{\max}}{t^{2}},$$

with t being the thickness of the specimen, and δ a function derived using finite element analysis. The Poisson’s ratio was taken as 0.314 as measured in ref. [15], which is typical for zirconias in the range of 3–6 mol% Y_2_O_3_ stabilization. The ratios of the specimen dimensions (thickness t and specimen radius R) to the support radius R_s_ and the Poisson’s ratio ν define the function δ [20]:

(4) $$\delta \left(\frac{t}{R_{s}},\frac{R}{R_{s}},\nu \right)\;=\;exp\left[0.697\left(1+\nu \right)\;-\;0.118ln\frac{t}{R_{s}}\;-\;0.728\sqrt[{4}]{\frac{Rt^{2}}{R_{s}^{3}}}\right]k_{1}k_{2},$$

where the support radius R_s_ = 2R_b_/$\sqrt{3}$ is formed by the three supporting balls of R_b_ = 4 mm. An effective diameter (D_eff_) can replace 2R for square-shaped plates [20]:

(5) $$D_{eff}\;=\;L\left(1.053\;-\;0.017\frac{tL}{R_{s}^{2}}\right),$$

with L being the length of the square sides. The increase in contact radius between the specimen and the loading ball during loading (at fracture) can be taken into account by the correction factor k_1_ [20]:

(6) $$k_{1}\;=\;1.0052\;+\;0.00063\;ln\left(R_{c}/R_{s}\cdot t/R_{s}\right)\;-\;0.5928\frac{{\left(R_{c}/R_{s}\right)}^{1.6756}}{{\left(t/R_{s}\right)}^{1.3523}},$$

(7) $$R_{c}\;=\;\sqrt[{3}]{\frac{3PR_{lb}}{4}\left(\frac{1\;-\;\nu^{2}}{E}\;+\;\frac{1\;-\;\nu_{lb}^{2}}{E_{lb}}\right),}$$

where R_c_ is the relative contact radius of the loading ball with the compression side of the specimen and E is the measured elastic modulus (subscript lb = hardened stainless steel 420 C loading ball, E_lb_ = 200 GPa, ν = 0.24). The additional factor k_2_ in Equation (2) corrects for the change in bending moment during the loading that arises from the shift in the contact point between the supporting balls and specimen [20]:

(8) $$k_{2}\;=\;1\;+\;\frac{\sqrt{3}}{2}\frac{P}{Et^{2}}\left[\left(1\;-\;\nu^{2}\right)\frac{R/R_{s}}{t/R_{s}}\left(0.0015\;-\;1.13\frac{1}{{\left(R/R_{s}\right)}^{2}}\right)\right]$$

The calculated strength values were evaluated using Weibull statistics using the maximum likelihood estimation to obtain the characteristic strength at 63.2% failure probability (scale factor, σ_0_) and the Weibull modulus (shape factor, m).

### 2.4. Fracture Toughness Testing

Oversized beams (height W, width B, length L of 4 mm × 3 mm × 25 mm plus 20% linear shrinkage) were cut from the blanks with the shortest axis (width B) aligned to the height of the blank, in order to sample the entire gradient profile by sawing the beams from different positions along the height, resulting in 7 different “planes” (see Figure 1). A total of 63 specimens were manufactured, nine samples out of seven planes for each material. Before sintering, the oversized beams relative to the corresponding planes (#1 to #7) were notched at the midsection with a thin 0.15 mm thick diamond disk using a custom notching machine [21,22] according to the Chevron-Notched-Beam (CNB) method, following the guidelines of ASTM C1421 [23]. Sintering was followed according to the manufacturer’s instructions, and any warping was ground to obtain plane parallelism. To protect the specimen against environmental moisture, thus minimizing slow crack growth effects [24], the specimens were dried in an oven at 150 °C together with a silicon oil bath, into which the specimens were immersed after 3 h. Specimens were tested at a loading rate of 0.05 mm/s in a fully articulated self-aligning custom testing jig [21,22] in four-point bending (4 PB) with 10–20 mm spans. The change in compliance during the test was measured on the specimen’s surface by means of the Speckle correlation approach (LaserXtens, Zwick/Roell, Ulm, Germany), thus enabling the detection of stable crack growth (crack pop-in) before instability to assure test validity. For valid specimens, the fracture toughness *K_Ic_* was calculated from the maximum force at fracture F_max_ [23]:

(9) $$K_{I,c}\;=\;\frac{F_{max}\left(S_{o}\;-\;S_{i}\right)}{BW^{3/2}}\cdot \frac{Y_{min}^{*}}{\sqrt{10^{3}}}$$

being S_o_ and S_i_ the outer and inner span lengths, respectively, with:

(10) $$Y_{min}^{*}=\frac{0.3874-3.0919\left(l_{0}/W\right)+4.2017\left(l_{1}/W\right)-2.3127{\left(l_{1}/W\right)}^{2}+0.6379{\left(l_{1}/W\right)}^{3}}{1-2.9686\left(l_{0}/W\right)+3.5056{\left(l_{0}/W\right)}^{2}-2.1374{\left(l_{0}/W\right)}^{3}+0.0130\left(l_{1}/W\right)}$$

where l_0_ is the distance between the bottom edge of the beam and the tip of the Chevron notch, and l_1_ an arithmetic mean of the notched segments on the sides of the beam, measured using a stereomicroscope (Discovery V8, Zeiss, Oberkochen, Germany) coupled with a digital camera (Axiocam 308, Zeiss, Oberkochen, Germany) and measuring software (ZenCore, Zeiss, Oberkochen, Germany). Our CNB testing procedures have been validated using a Standard Reference Material and shown consistency for zirconia and other ceramic-based materials [25]. The four-point bending with 10–20 mm spans has been shown to provide equivalent test accuracy to 20–40 mm spans [22,26].

## 3. Results and Discussion

### 3.1. QXRD

**Figure 2:**
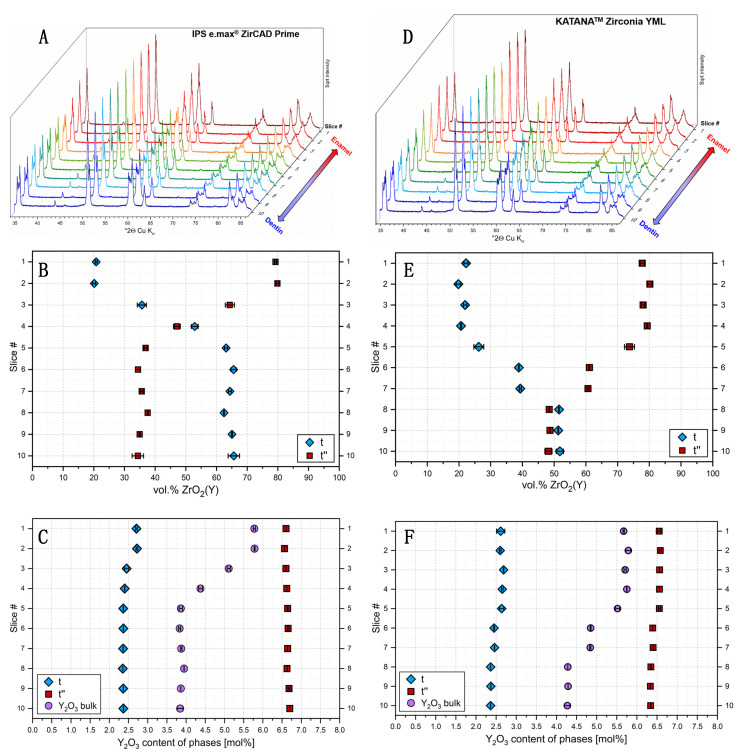
(**A**,**D**) Diffraction patterns, (**B**,**E**) volume fractions of t and t″ and (**C**,**F**) Y_2_O_3_ contents of t, t″ and the bulk samples of the ten slices cut from IPS e.max^®^ ZirCAD Prime and KATANA^TM^ Zirconia YML.

For both KATANA^TM^ Zirconia YML and IPS e.max^®^ ZirCAD Prime, a change in the diffraction patterns was directly evident from the enamel to the dentin section (Figure 2A). While the general position of the reflections remained the same, the height ratio between the different reflections changed.

For all slices, Rietveld refinement was successfully accomplished by using two tetragonal ZrO_2_(Y) structures, corresponding to the phases t and t″, as in [15] (the refinement is shown in Figure 5a,b in [15]). It is evident that the diffraction pattern of slice #1 looks very similar to that of the 5Y-TZP material Cercon^®^ xt, while that of slice #10 is nearly identical to that of the 3Y-TZP material Cercon^®^ ht [15]. Further addition of a structure model for cubic ZrO_2_(Y) did not result in a significant improvement of the fit. Therefore, only the two tetragonal phases t and t″ were considered in the following quantitative evaluation.

In e.max^®^ ZirCAD Prime, a high fraction of t″ phase, 78 to 80 vol.%, was present in the first two slices, while a constant decrease in t″ fraction was detected between the second and the fifth slice. After that, a plateau with lower t″ fractions between 34 and 37 vol.% was reached (Figure 2B). The development of the Y_2_O_3_ bulk content showed a similar behavior (Figure 2C) as the t″ quantities. The t phase contained between 2.35 and 2.7 mol% Y_2_O_3_, with a slight decrease after slice #2.

In contrast, the Y_2_O_3_ contents of t″ were remarkably higher with values between 6.5 and 6.7 mol%. In KATANA^TM^ Zirconia YML, an initial plateau with t″ fractions between 77 and 80 vol.% was observed. Between slices #4 and #8, a stepwise decrease in t″ fraction occurred, until a plateau with around 48 vol.% t″ was reached (Figure 2B). A similar behavior was observed for the Y_2_O_3_ bulk content. The Y_2_O_3_ content of phase t was between 2.3 and 2.7 mol%, while t″ contained between 6.3 and 6.6 mol% Y_2_O_3_. A slight decrease in the Y_2_O_3_ content from slice #1 to #10 was observed for both t and t″.

According to the manufacturer’s description, the material IPS e.max^®^ ZirCAD Prime contains a gradient (transition) zone between layers of 3Y-TZP (dentin) and 5Y-TZP (enamel/incisal). The material has an overall thickness of 16 mm. The upper 3 mm correspond to the enamel layer [27]. Indeed, the bulk Y_2_O_3_ content, as well as the content of Y_2_O_3_-rich t″ phase, were the highest in the first two slices, produced from the enamel zone (Figure 2B,C). The transition zone is 4 mm in thickness [27], thus ranging until slice #5. A decrease in Y_2_O_3_ bulk content and t″ fraction was observed exactly within this region. The underlying dentin zone is well represented by the plateau with low Y_2_O_3_ and t″ content.

In contrast, KATANA^TM^ Zirconia YML is reported to be composed of four distinct layers, namely “enamel”, “body 1”, “body 2” and “body 3”; its overall thickness is 18 mm [27]. The enamel layer, 6.3 mm in thickness, reaches until slice #4 and corresponds to the plateau with high t″ fraction and bulk Y_2_O_3_ content. In the following slices, body 1 (2.7 mm) and body 2 (2.7 mm) can be observed as distinct intermediate layers with stepwise decreasing t″ weight fraction from surface to bottom, until a final plateau with even lower t″ fraction is reached within body 3.

In summary, it can be stated that the observed scheme of variation in t″ weight fraction and calculated Y_2_O_3_ bulk content from enamel to dentin part is well in accordance with the manufacturer’s description for both materials.

Furthermore, it is evident that the calculated Y_2_O_3_ contents of both t and t″ are very similar in both samples investigated, and they further show only little variation throughout the samples. For phase t, the Y_2_O_3_ contents varied between 2.3 and 2.7 mol%, while t″ contained between 6.3 and 6.7 mol% Y_2_O_3_ in both samples. In the different non-graded materials characterized in [15], Y_2_O_3_ contents between 2.3 and 3.1 mol% were obtained for t and between 6.1 and 7.2 mol% for t″. Thus, the data were even comparable between the different studies, and it seems that both t and t″ tend to have a rather constant Y_2_O_3_ content, independent of the overall material composition.

The calculated Y_2_O_3_ contents from this study for both slice #1 and #10 were further compared with the corresponding XRF data presented by Inokoshi et al. [27] (Table 2). It is evident that for both IPS e.max^®^ ZirCAD Prime and KATANA^TM^ Zirconia YML, the calculated Y_2_O_3_ contents of the enamel part agree very well with the XRF data from [27], while the obtained contents from the dentin part were slightly overestimated within this study (Table 2). This might be either due to differences in composition between the samples used in the different studies, or some error in the estimation of the Y_2_O_3_ contents within this study, which is dependent on both wt.% fractions and lattice parameters obtained by Rietveld refinement, as well as the accuracy of the equation presented by Miller et al. [28]. However, it was proven that the data indirectly calculated from XRD are at least close to the data obtained by direct chemical analysis (XRF).

Furthermore, it can be noticed that the t″ weight fraction in the enamel part is very similar for both materials, while in the dentin zone, the t″ fraction is more than 10 wt.% lower in IPS e.max^®^ ZirCAD Prime. This is in accordance with the lower Y_2_O_3_ bulk content of the dentin part in this sample.

The results indicate that in both IPS e.max^®^ ZirCAD Prime and KATANA^TM^ Zirconia YML, the incisal zone is actually composed of a 5Y-TZP material, though the Y_2_O_3_ content appears to be slightly above 5 mol%. While the dentin part can be assigned as 3Y-TZP in IPS e.max^®^ ZirCAD Prime, it should be rather addressed as 4Y-TZP in KATANA^TM^ Zirconia YML, due to the higher Y_2_O_3_ bulk content.

### 3.2. Mechanical Properties

**Figure 3:**
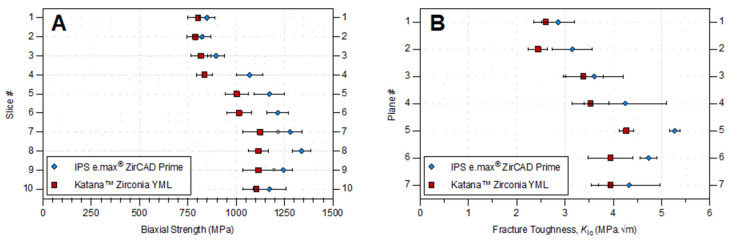
Average and standard deviations (error bars) of (**A**) biaxial strength and (**B**) fracture toughness according to the slice# and plane# for IPS e.max^®^ ZirCAD Prime and KATANA^TM^ Zirconia YML.

The compositional gradient in yttrium oxide, confirmed by XRD analysis, was directly reflected in the mechanical behavior of both multilayer zirconia systems. A clear trend along the height of the blanks between Y_2_O_3_ content and fracture toughness was observed, consistent with the stabilizing influence of yttria on the tetragonal phases t and t″, as previously reported by [29,30]. The upper slices (#1) of IPS e.max ZirCAD Prime and KATANA Zirconia YML exhibited higher Y_2_O_3_ concentrations and a predominance of the t″ phase, around 6.5–6.65 mol%, whereas the basal slices (#10) displayed lower Y_2_O_3_ contents and a dominance of the transformable tetragonal phase of approximately 2.37 mol%. This compositional “gradient” was mirrored by a gradual increase in both fracture toughness and flexural strength from the enamel-like to the dentin-like regions (Figure 3, Table 3 and Table 4), in line with [1,27].

In ZirCAD Prime, fracture toughness increased from 2.86 ± 0.34 MPa$\sqrt{m}$ in plane #1 to 4.33 ± 0.64 MPa$\sqrt{m}$ in plane #7, whereas KATANA YML increased from 2.6 ± 0.24 MPa$\sqrt{m}$ to 3.94 ± 0.40 MPa$\sqrt{m}$. The inverse relationship between yttria concentration and toughness confirms that higher Y_2_O_3_ contents reduce the potential for stress-induced transformation toughening due to greater stabilization of the t″ phase (sometimes reportedly cubic) [4,31,32]. These findings correspond closely with those of Strasser et al. [1] who demonstrated higher cubic phase fractions and lower strength in 5Y-TZP enamel layers compared to 3Y-TZP dentin layers, and with the data of Inokoshi et al. [27], who observed an analogous correlation between phase composition, yttria content, and flexural strength.

The biaxial flexural strength results supported this phase-dependent trend. For ZirCAD Prime, strength increased from 849 MPa in slice #1 to 1173 MPa in slice #10; for KATANA^TM^ YML, strength increased from 803 MPa to 1104 MPa (Table 3). These results align with the strength-gradient behavior reported by previous studies [1,27], as well as with previous studies on multilayered zirconia, demonstrating that flexural strength increases progressively from incisal to cervical layers as Y_2_O_3_ content decreases [22,29,33]. The trend reinforces that the introduction of nearly cubic t″ phase enhances translucency at the expense of mechanical properties, as discussed in [32,34].

When comparing the best-performing slices of both materials, ZirCAD Prime exhibited slightly higher flexural strength compared with KATANA YML. Such a comparison regarding fracture toughness again favored ZirCAD Prime. The difference between analogous yttria concentrations in the two materials is most probably due to the different powders used by the companies, specifically the fabrication process utilized to stabilize the zirconia primary nanoparticles within the granulates. However, the discrepancies in mechanical properties increase toward the dentin layer. Regarding the gradient profile of yttria stabilization, ZirCAD Prime is considerably lower in the dentin layer (about 3.8 mol%) than in KATANA YML (about 4.3 mol%); Inokoshi et al. [27] observed a similar difference between the two materials, despite their concentration at the dentin layer being lower than our measurements (see Table 2). The observed differences agree with [15], who emphasized that the mechanical response of modern Y-TZP ceramics is strongly governed by local Y_2_O_3_ content and the associated balance of t/t″/c phases.

In KATANA YML, the stepwise profile in yttria concentration can be seen very clearly reflected in the mechanical behavior, especially by the strength measurements, which is consistent with the more moderate gradient identified in ref. [27]. In ZirCAD Prime though, the more continuous transition zone localized in the region of slice #3 and #4 are well reflected in both mechanical assessments, but the dentin layer showed not to be entirely constant from slice #5 to #10 (plane #4 to #7), but increasing further in slices #7 and #8 (planes #5 and #6). Differences in strength can be well argued in terms of the compaction and size distribution of pressing defects, which can be heterogeneous along the height of a pressed blank and exhibit higher defect sizes at the surfaces [2]. The Weibull moduli further supported that conjecture. For both systems, the Weibull modulus (m) increased toward the lower slices, indicating higher structural reliability and reduced flaw sensitivity in the predominantly tetragonal regions [15]. ZirCAD Prime reached m~8–11 in the mid-to-lower slices, whereas KATANA YML exhibited slightly lower values overall (m~6–9), reflecting wider defect size distributions. Similar trends were described by [1,15], who associated lower m values with phase heterogeneity and microstructural transitions in gradient zirconia.

Overall, these results demonstrate that the Y_2_O_3_ gradient determined by XRD analysis is directly reflected in the mechanical performance of both systems. The inverse relation between yttria content and fracture toughness, the concurrent increase in flexural strength toward 3Y-rich layers, and the material-specific differences in reliability confirm that the mechanical behavior of multilayer zirconia is dictated by the profile of the yttria gradient [1,15,27,29,30,32]. These findings are consistent with the current literature and emphasize that the mechanical properties of gradient zirconia must be interpreted in a layer-specific context that reflects the local Y_2_O_3_ distribution and phase composition. Nonetheless, the limitations of the chosen study design must be acknowledged. Owing to the selected plane location, the loss of material during the sawing process, and the absence of comparative statistical analyses, the findings should be interpreted as primarily descriptive in nature. The distinct grading approach identified and confirmed in this study should therefore be incorporated into future research, specifically designed to enable a direct comparison of the mechanical properties of both materials rather than focusing solely on their characterization.

## 4. Conclusions

This study demonstrated that multilayer zirconias exhibit distinct manufacturer-specific gradient profiles, which can be reliably characterized using quantitative X-ray diffraction and Rietveld refinement. Both IPS e.max^®^ ZirCAD Prime and KATANA™ Zirconia YML showed systematic variations in Y_2_O_3_ content and phase composition (t and t″) along the blank height, consistent with their reported multilayer designs. Mechanical testing confirmed a clear structure–property relationship, where regions with higher yttria content and increased t″ fractions exhibited reduced flexural strength and fracture toughness, while deeper regions showed improved mechanical performance associated with a higher contribution of transformable tetragonal phase. Clinically, these findings indicate that the positioning of restorations within multilayer blanks may influence fracture risk, particularly in load-bearing regions where high-translucency layers may compromise mechanical reliability. Therefore, multilayer zirconias should be considered functionally graded biomaterials requiring layer-aware material selection and restoration design.

## Figures and Tables

**Table 1:** ICSD structures used for refinement of ZrO_2_(Y) samples.

Phase	ICSD #	Space Group	Authors
ZrO_2_(Y)	89428	Tetragonal P4_2_/nmc	Wang et al. (1999) [16]
ZrO_2_(Y)	89429	Cubic Fm-3m	Wang et al. (1999) [16]

**Table 2:** Bulk Y_2_O_3_ contents of IPS e.max^®^ ZirCAD Prime and KATANA^TM^ Zirconia YML, either calculated from XRD data within this study, or determined by XRF analysis [27]; the data from slice #1 were taken for enamel and those from slice #10 for dentin.

Y_2_O_3_ Bulk [mol%]	Prime-Dentin	Prime-Enamel	YML-Dentin	YML-Enamel
This study	3.84 ± 0.08	5.78 ± 0.03	4.27 ± 0.06	5.67 ± 0.02
Inokoshi et al. [27]	3.14 ± 0.02	5.59 ± 0.04	3.86 ± 0.01	5.60 ± 0.04

**Table 3:** Flexural strength values (σ_0_) and m modulus for both tested materials with the respective 90% confidence interval.

	IPS e.max^®^ ZirCAD Prime		Katana™ Zirconia YML	
Slice #	σ_0_ [90% C.I.] (MPa)	*m* Modulus [90% C.I.]	σ_0_ [90% C.I.] (MPa)	*m* Modulus [90% C.I.]
1	849.42 [807–893]	7.9 [5.8–10.7]	802.87 [749–861]	5.9 [4.3–8.1]
2	821.93 [774–873]	6.3 [4.7–8.4]	788.68 [745–834]	7.1 [5.2–9.6]
3	895.33 [851–942]	7.8 [5.8–10.6]	816.96 [765–873]	6.2 [4.5–8.5]
4	1068.93 [1001–1142]	5.7 [4.3–7.7]	836.4 [796–879]	8 [6.0–10.9]
5	1171.9 [1093–1257]	6.2 [4.4–8.6]	1002.78 [942–1067]	6.6 [4.8–9.0]
6	1215.05 [1159–1273]	8.7 [6.4–11.9]	1014.76 [950–1085]	6.3 [4.6–8.8]
7	1279.94 [1220–1343]	8.4 [6.2–11.6]	1123.49 [1036–1220]	5.3 [3.8–7.4]
8	1337.79 [1290–1387]	10.8 [8.0–14.5]	1115.79 [1063–1171]	9.5 [6.7–13.6]
9	1245.25 [1200–1292]	10.7 [7.9–14.6]	1113.78 [1036–1198]	6.1 [4.4–8.6]
10	1172.54 [1088–1264]	5.5 [4.0–7.5]	1104.09 [1036–1177]	6.8 [4.9–9.5]

**Table 4:** Mean values and standard deviations for the fracture toughness of the tested materials, IPS e.max^®^ ZirCAD Prime and KATANA™ Zirconia YML, for each individual slice.

	IPS e.max^®^ ZirCAD Prime	Katana™ Zirconia YML
Plane #	$K_{\mathrm{Ic}}\;\pm \;S.D.\;(\mathrm{MPa}\sqrt{m})$	$K_{\mathrm{Ic}}\;\pm \;S.D.\;(\mathrm{MPa}\sqrt{m})$
1	2.86 ± 0.34	2.60 ± 0.24
2	3.15 ± 0.41	2.44 ± 0.20
3	3.61 ± 0.59	3.38 ± 0.42
4	4.25 ± 0.85	3.53 ± 0.38
5	5.27 ± 0.11	4.27 ± 0.15
6	4.73 ± 0.18	3.94 ± 0.46
7	4.33 ± 0.64	3.94 ± 0.40

## Data Availability

The original contributions presented in this study are included in the article. Further inquiries can be directed to the corresponding author.
